# The Prevalence of Musculoskeletal Injuries Among Pilgrims During the 2023 Hajj Season: A Cross-Sectional Study

**DOI:** 10.7759/cureus.56754

**Published:** 2024-03-23

**Authors:** Ghidaa A Alghamdi, Faisal A Alghamdi, Renad M Almatrafi, Arwa Y Sadis, Rozan A Shabkuny, Saad A Alzahrani, Mohammed Q Alessa, Waleed A Hafiz

**Affiliations:** 1 Department of Medicine and Surgery, College of Medicine, Umm Al-Qura University, Makkah, SAU; 2 Department of Medicine, College of Medicine, Umm Al-Qura University, Makkah, SAU; 3 Department of Medicine, Al-Noor Specialist Hospital, Makkah, SAU

**Keywords:** overcrowding, fatigue, ankle, knee pain, back pain, pilgrims, hajj season, musculoskeletal injuries

## Abstract

Background

Hajj, the annual Islamic pilgrimage, brings together over two million pilgrims in the city of Makkah to participate in a series of rituals. Given the physically demanding nature of the Hajj, pilgrims are susceptible to musculoskeletal (MSK) injuries and exhaustion. MSK pain and injuries are frequent occurrences among pilgrims, necessitating an assessment of the scope of this issue. Therefore, the primary objective of this study was to determine the prevalence of MSK injuries among pilgrims during the 2023 Hajj season.

Methods

This is a cross-sectional questionnaire-based study that was conducted in the city of Makkah, Saudi Arabia, during the 2023 Hajj season.

Results

A total of 463 pilgrims were included in the analysis. The most frequently reported types of injuries were muscular injuries (169, 45.4%), primarily characterized by pain (99, 58.6%), muscle spasms (55, 32.5%), and muscle tears (eight, 4.7%). The second most commonly reported MSK injury was bony injuries (97, 26.1%), which included fractures, followed by 79 cases (21.2%) of joint injuries, predominantly featuring pain (69, 87.3%) and joint prolapse (10, 12.7%). Notably, 27 pilgrims (7.3%) suffered from ligament injuries, including tears. Regarding the mechanisms or causes of these MSK injuries, the most frequently reported factors were fatigue (206, 55.4%), falls (76, 20.4%), crowding (34, 9.1%), accidents (30, 8.1%), and the use of wheelchairs (14, 3.8%). Additionally, it is noteworthy that muscular injuries were more prevalent among all age groups, particularly among young-aged pilgrims, while joint injuries were more common among elderly pilgrims.

Conclusion

MSK injuries are prevalent among pilgrims, with muscular injuries being the most frequently encountered. This underscores a noteworthy public health concern that necessitates attention from the Ministry of Health of Saudi Arabia.

## Introduction

The musculoskeletal (MSK) system is one of the human body's systems that functions to provide support and facilitate easy movement. This system comprises crucial structures, including the bones, muscles, cartilage, joints, ligaments, and soft tissues. With distinct functions and unique morphology for each of these structures, they collaborate to shield the body from harm and maintain its stability [1].

Injuries to these structures are acknowledged as a major health issue in the United States. Approximately 161,269 people lost their lives to injuries in 2002 [2]. All the participants in one publication discussing the epidemiology of MSK injuries were 5,028 males and 1,285 females; the lower extremities accounted for almost 66% of their injuries, with the knee being the most commonly injured joint [3].

Hajj is an annual Islamic ritual that unites over two million pilgrims in the city of Makkah to engage in a series of sacred practices and requires significant physical demands from the participants. One hundred eighty-four nations gather to perform the Hajj at Makkah, Saudi Arabia's holiest site, known as "Haram" [4]. All Muslims who are in good physical and financial health have a once-in-a-lifetime responsibility to do the Hajj or pilgrimage to the Holy Mosque in Makkah [5]. According to research done among the Algerian population, 2.2 billion Muslims are expected to exist by 2030 because it is a fast-growing religion [6]. This strenuous endeavor exposes pilgrims to the risk of MSK injuries and exhaustion. Pilgrims have historically been more susceptible to certain illnesses and traumas, such as respiratory, especially viruses [7]; gastrointestinal; and MSK injuries [8]. MSK pain and injuries are commonplace among these pilgrims and represent a prevalent category of noncommunicable diseases during Hajj [9].

Literature reviews have demonstrated that trauma injuries resulting from incidents such as falls, slips, stampedes, and traffic accidents account for 27 (9.4%) of hospital admissions and 28 (6.4%) of intensive care unit (ICU) admissions in Makkah hospitals during the Hajj season [9]. Trauma sustained during the Hajj is a legitimate surgical issue that requires further study. During the Hajj period, the most common surgical cases were those involving orthopedics and neurosurgery [10]. Furthermore, trauma incidents constitute a critical factor associated with mortality during the Hajj season [11].

A significant proportion of MSK injuries are attributed to the large gatherings and the rigorous physical demands imposed on pilgrims. These demands often entail walking an average of 5-15 km daily, coupled with frequent travel and accommodations in tents. It is noteworthy that the most frequent injuries occur during specific rituals, such as Tawaf, Saee, and Ramy al-Jamarat [9,12]. A comprehensive study involving 1,715 pilgrims demonstrated that the estimated prevalence of falls was 236 (13.76%), with a higher incidence in females and pilgrims aged over 50. The overall prevalence of MSK pain was 1,190 (80.46%), with ankle/foot pain being the most prevalent, followed by leg pain, lower back pain, and knee pain [12,13]. Another study mentioned that 61 (31%) of the 197 pilgrims from 21 different nations that took part in this study had diabetes. Two of the most frequent ailments seen from the total pilgrims were erythema (44, 25%) and blisters (67, 34%) [14].

While numerous studies have investigated health issues among pilgrims, with a focus on infectious diseases and other ailments, including cardiovascular diseases, neurological disorders, injuries, gastrointestinal problems, diabetes, heat-related conditions, and dermatological diseases, the frequency of MSK injuries among pilgrims performing Hajj and any potential contributing factors have yet to be addressed. The aim of this study was to determine the prevalence of MSK injuries among pilgrims during the 2023 Hajj season.

## Materials and methods

Study design

This was a cross-sectional questionnaire-based study conducted in the city of Makkah, Saudi Arabia, during the 2023 Hajj season. The participants were informed of the study's objectives, and their verbal consent was obtained for their participation. It is essential to emphasize that all information and data collected were strictly utilized for the sole purpose of this study.

Study population

The 2023 Hajj season was expected to attract approximately two million pilgrims. Our study's target population was determined by specific inclusion and exclusion criteria (Table 1).

**Table 1:** Inclusion and exclusion criteria of the study

Inclusion criteria	Exclusion criteria
Adult pilgrims aged ≥18 years, both males and females	Pilgrims with incomplete data
Arabic and non-Arabic language speakers	Individuals younger than 18 years of age

Study procedures

The study was executed through an online questionnaire constructed using Google Forms (Google, Inc., Mountain View, CA). Data collection took place at various pilgrimage sites in Makkah, as well as in the pilgrim camps, Al-Noor Specialist Hospital, and Mina Al-Jisr Hospital, employing a probability sampling technique. The researchers selected the participants systematically based on their pilgrim status. In cases where the patients encountered difficulties comprehending any question, the researchers followed a predetermined script to elucidate any aspects of the survey, thereby minimizing any potential misunderstandings. The questionnaire was divided into four distinct sections: The first section encompassed demographic data, the second assessed the participants' chronic illnesses and any previous MSK injuries, the third gathered details concerning any MSK injuries experienced by the participant, and the final section focused on the participants' opinions regarding preventive measures that could be employed to avert MSK injuries during the performance of Hajj rituals.

Data collection and management

Data collection was facilitated through the use of an electronic questionnaire. Subsequently, the collected data were automatically input into an Excel spreadsheet (Microsoft Corp., Redmond, WA). Following this initial data compilation, the dataset was exported to the Statistical Package for Social Sciences (SPSS) software (IBM SPSS Statistics, Armonk, NY) for the purpose of statistical analysis.

Sample size determination

The minimum required sample size for this study was determined using OpenEpi version 3.0, taking into account the following parameters: a population size estimated to be approximately 1,845,045 (as reported by the General Authority for Statistics in 2023), a confidence interval (CI) set at 95%, and an anticipated frequency of 50%. Based on these factors, the calculated sample size was 385 participants.

Statistical analysis plan

The data were analyzed using a t-test to compare the mean difference across two different groups. A one-way analysis of variance (ANOVA) was used to compare across more than two groups. Chi-square and Fisher's exact tests were used to compare the categorical data. Numeric data were presented as mean ± SD or as median and interquartile range according to the type of distribution of each variable. A p-value greater than 0.05 was considered statistically significant. For categorical variables, frequencies and percentages were used.

Ethical part and confidentiality

Prior to responding to the questionnaire, each participant provided verbal consent. Ethical approval for the study was granted by the Biomedical Research Ethics Committee of Umm Al-Qura University (approval number: HAPO-02-K-012-2023-09-1717). To maintain confidentiality, the participants were assigned serial codes rather than using their names or personal identification numbers. Access to the data was strictly limited to the investigators, ensuring privacy and data security. All study documents were securely locked, submitted, and stored in the principal investigator's office, adhering to ethical and privacy protocols.

Data analysis

The collected data underwent a structured process that included review and subsequent input into the Statistical Package for Social Sciences (SPSS) version 21 (IBM SPSS Statistics, Armonk, NY). All statistical analyses conducted in the study were two-tailed, with an alpha level of 0.05, considering results as statistically significant when the p-value was less than or equal to 0.05.

Descriptive analysis was performed by presenting frequency distributions and percentages for various study variables, which encompassed the personal data of pilgrims, their medical information, and their nationality. MSK disorders were tabulated, and signs, symptoms, and recommended methods for preventing MSK injuries were graphically represented.

Cross-tabulation was employed to illustrate associations between factors and MSK injuries among pilgrims. This was accomplished using the Pearson chi-square test for statistical significance, and in cases where frequency distributions were small, the exact probability test was applied. A correlation analysis was also conducted to examine the nature of the relationships between various study variables. These analytical techniques were chosen to comprehensively explore the data and derive meaningful insights from the study.

## Results

A total of 463 pilgrims were included. The participants' ages ranged from 18 to 101 years, with a mean age of 50.3 ± 15.8 years. Exactly 238 (51.4%) of the pilgrims were males, and 363 (78.4%) were non-Saudi. Regarding educational level, 155 (33.5%) had a university level of education, 100 (21.6%) had a below-secondary level of education, and 25 (5.4%) had a post-graduate degree. In terms of chronic diseases, 154 (33.3%) were hypertensive, 145 (31.3%) were diabetic, 53 (11.4%) had cardiac diseases, and 193 (41.7%) had no chronic health problems (Table 2).

**Table 2:** Personal characteristics of the study pilgrims attending the 2023 Hajj season

Personal data	Number	Percentage
Age in years		
<30	53	11.4%
30-39	65	14.0%
40-49	102	22.0%
50-59	109	23.5%
60+	134	28.9%
Gender		
Male	238	51.4%
Female	225	48.6%
Nationality		
Saudi	100	21.6%
Non-Saudi	363	78.4%
Educational level		
Did not answer	90	19.4%
Below secondary	100	21.6%
Secondary	93	20.1%
University graduate	155	33.5%
Post-graduate	25	5.4%
Chronic diseases		
None	193	41.7%
Hypertension (HTN)	154	33.3%
Diabetes mellitus (DM)	145	31.3%
Others	59	12.7%
Cardiac disease	53	11.4%
Strokes	16	3.5%
Cancer	8	1.7%

**Figure 1:**
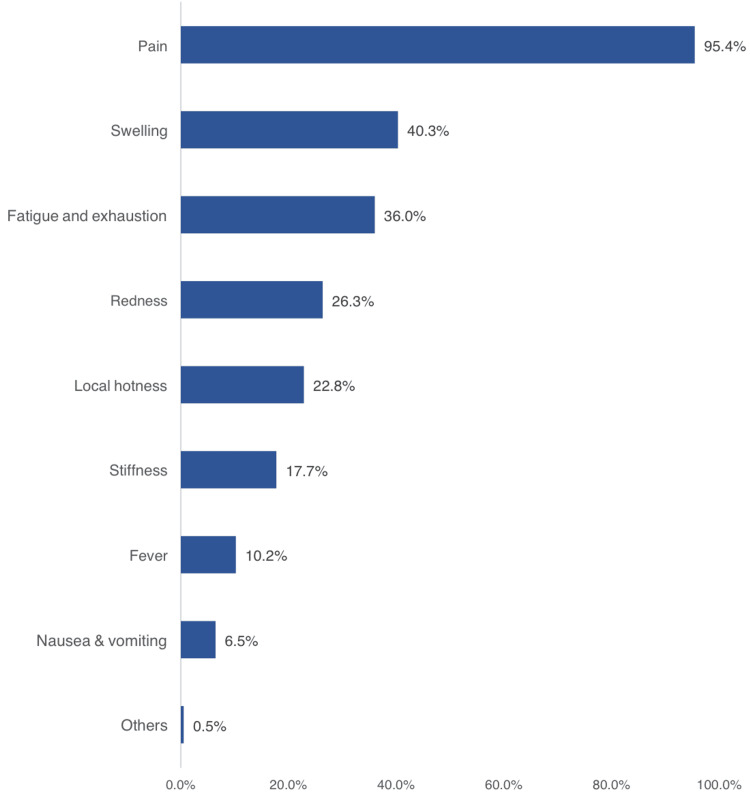
Signs and symptoms of reported MSK injuries among pilgrims attending the 2023 Hajj season MSK: musculoskeletal

Table 3 shows that exactly 226 (48.8%) suffered from MSK injuries, and 372 (80.3%) experienced MSK injuries during the current Hajj season. The most frequently reported types of injuries were muscular injuries (169, 45.4%), primarily pain (99, 58.6%), spasm (55, 32.5%), and tear (eight, 4.7%). The second most reported MSK injury was bony injuries (97, 26.1%), including fractures. Seventy-nine (21.2%) individuals reported joint injuries, mainly pain (69, 87.3%) and prolapse (10, 12.7%). Twenty-seven (7.3%) pilgrims suffered from ligament injuries, including tears. Regarding the mechanisms (causes) of MSK injuries, the most commonly reported causes included fatigue (206, 55.4%), falls (76, 20.4%), crowding (34, 9.1%), accidents (30, 8.1%), and wheelchair-related injuries (14, 3.8%). The associated signs and symptoms of reported MSK injuries among pilgrims attending the 2023 Hajj season are shown in Figure 1.

**Table 3:** MSK disorders among pilgrims attending the 2023 Hajj season

Musculoskeletal (MSK) disorders	Number	Percentage
Have you ever suffered a musculoskeletal (MSK) injury?		
Yes	226	48.8%
No	130	28.1%
Do not remember	107	23.1%
Did you suffer a musculoskeletal (MSK) injury during the current Hajj season?		
Yes	372	80.3%
No	91	19.7%
Type of injury		
Muscular-tendon	169	45.4%
Bony	97	26.1%
In the joints	79	21.2%
In the ligaments	27	7.3%
If bony		
Fracture	97	100.0%
If muscular		
Pain	99	58.6%
Tear	8	4.7%
Spasm	55	32.5%
Inflammation	7	4.1%
If ligament		
Tear	27	100.0%
If joint		
Pain	69	87.3%
Prolapse	10	12.7%
Mechanisms (causes) of musculoskeletal (MSK) injury		
Fatigue	206	55.4%
Fall	76	20.4%
Crowding	34	9.1%
Accident	30	8.1%
Wheelchair	14	3.8%
Others	12	3.2%

**Figure 2:**
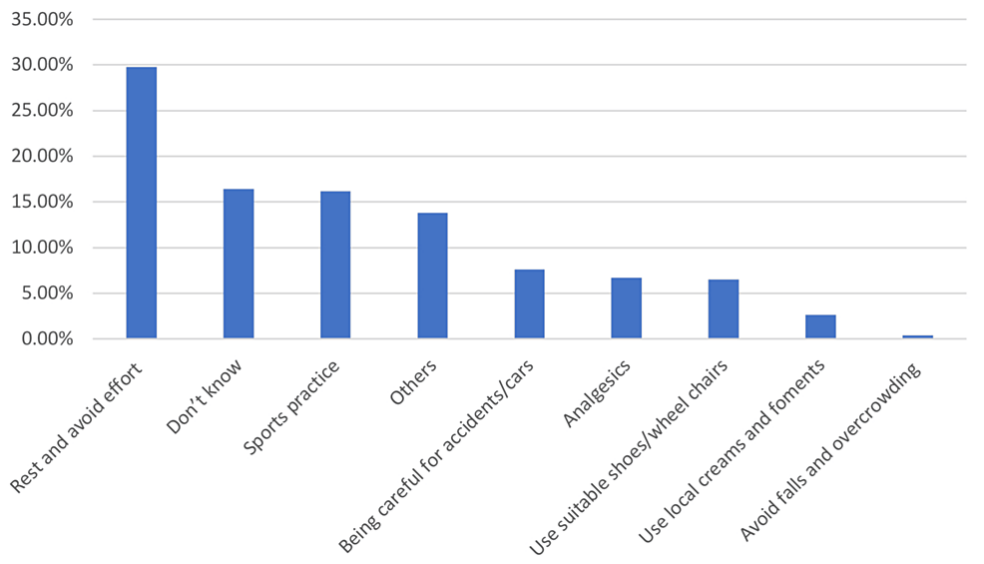
Suggested methods to prevent MSK injuries among pilgrims attending Hajj season MSK: musculoskeletal

Figure 2 shows the suggested methods to prevent MSK injuries among pilgrims attending the Hajj season. The most frequently reported methods were taking rest and avoiding strenuous efforts (138, 29.8%), engaging in sports activities (75, 16.2%), being more cautious (35, 7.6%), using analgesics (31, 6.7%), wearing appropriate shoes and using wheelchairs (30, 6.5%), and avoiding falls and overcrowding (two, 0.4%).

**Figure 3:**
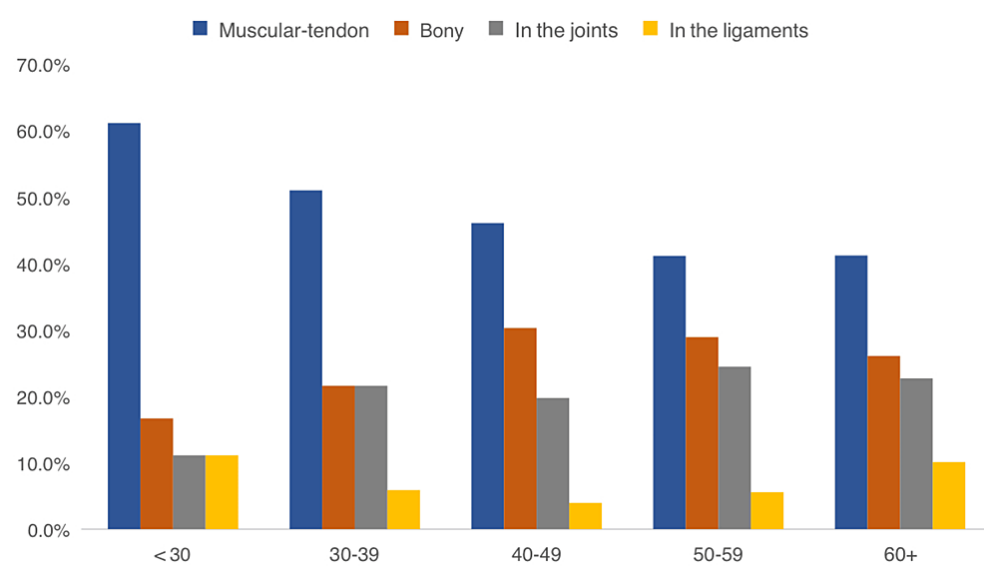
Distribution of the type of injury by pilgrim's age

Table 4 shows the factors associated with MSK injuries among pilgrims attending the Hajj season. A total of 119 (88.8%) pilgrims aged 60 years or older had MSK injuries during Hajj, compared to 36 (67.9%) of those aged less than 30 years, with statistically significant differences (P = 0.007). Additionally, 309 (85.1%) of non-Saudi pilgrims experienced MSK injuries, in contrast to 63 (63%) of Saudi pilgrims (P = 0.001). A total of 227 (82.8%) pilgrims with chronic health problems experienced MSK injuries during Hajj, compared to 145 (76.7%) of those without (P = 0.049). Muscular injuries were the most frequently reported among all age groups, especially among younger pilgrims, while joint injuries were more common among older pilgrims, as shown in Figure 3.

**Table 4:** Factors associated with MSK injuries among pilgrims attending the Hajj season ^$^Exact probability test *P < 0.05 (significant) MSK: musculoskeletal

Factors	Did you suffer an MSK injury during the current Hajj season?				P-value
	Yes		No		
	Number	Percentage	Number	Percentage	
Age in years					0.007*
<30	36	67.9%	17	32.1%	
30-39	51	78.5%	14	21.5%	
40-49	76	74.5%	26	25.5%	
50-59	90	82.6%	19	17.4%	
60+	119	88.8%	15	11.2%	
Gender					0.176
Male	197	82.8%	41	17.2%	
Female	175	77.8%	50	22.2%	
Nationality					0.001*
Saudi	63	63.0%	37	37.0%	
Non-Saudi	309	85.1%	54	14.9%	
Educational level					0.830^$^
Did not answer	73	81.1%	17	18.9%	
Below secondary	80	80.0%	20	20.0%	
Secondary	72	77.4%	21	22.6%	
University graduate	125	80.6%	30	19.4%	
Post-graduate	22	88.0%	3	12.0%	
Chronic diseases					0.049*
No	145	76.7%	44	23.3%	
Yes	227	82.8%	47	17.2%	

**Figure 4:**
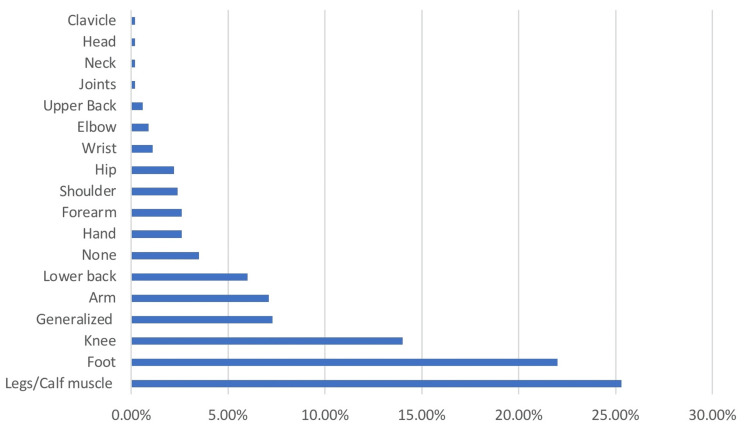
Site of pain among pilgrims attending the 2023 Hajj season

Table 5 shows that the most reported sites included the legs/calf muscles (117, 25.3%), foot (102, 22%), knee (65, 14%), arm (33, 7.1%), lower back (28, 6%), forearm (12, 2.6%), and hand (12, 2.6%). Thirty-four (7.3%) pilgrims had generalized pain, and 16 (3.5%) reported no site (Figure 4).

**Table 5:** Site of pain among pilgrims attending the 2023 Hajj season

Site of pain	Number	Percentage
Legs/calf muscle	117	25.3%
Foot	102	22.0%
Knee	65	14.0%
Generalized	34	7.3%
Arm	33	7.1%
Lower back	28	6.0%
None	16	3.5%
Forearm	12	2.6%
Hand	12	2.6%
Shoulder	11	2.4%
Hip	10	2.2%
Lower back up to the foot	7	1.5%
Wrist	5	1.1%
Elbow	4	0.9%
Upper back	3	0.6%
Clavicle	1	0.2%
Head	1	0.2%
Neck	1	0.2%
Joints	1	0.2%

## Discussion

In the 2023 Hajj season (1445 Hijri year), a total of 463 pilgrims participated. The principal objective of our study was to ascertain the prevalence of MSK injuries among pilgrims during the 2023 Hajj season. According to our findings, a substantial likelihood existed that pilgrims would experience MSK discomfort during the Hajj. The most frequently reported type of pain was muscular, encompassing discomfort in the foot, leg, and lower back regions. Our study revealed that the most commonly reported types of injuries were muscular (pain and spasm), bony (fracture), joint (pain and prolapse), and ligament tears, respectively. Muscular injuries exhibited the highest frequency across all age groups, whereas joint injuries were more prevalent among older individuals. This study suggests a significant association between the prevalence of MSK injuries and factors related to age and nationality. Notably, advanced age and non-Saudi nationality were associated with a higher percentage of MSK injuries, potentially attributed to the fact that a considerable portion of the participants were older (363 {78.4%} of whom were non-Saudi). As reported by patients, the most commonly suggested methods to prevent MSK injuries included resting, avoiding strenuous physical activity, being cautious, taking analgesics, wearing appropriate footwear, and avoiding overcrowded situations.

In a study conducted in July 2023, it was noted that 264 (50%) of their participants during the 2022 Hajj season fell within the age range of 33-48 years [15]. In contrast, our study found that 243 (52.4%) of our participants were between 50 and 101 years old. This difference might be attributed to variations in data collection locations. Our study included data from not only hospitals but also various pilgrim gathering sites, where younger participants could access medical attention and potentially be transferred to hospitals more easily [15]; unlike previous studies where a significant majority of patients were male (264, 70%-80%) [15,16], our study observed nearly equal percentages for both genders, with 238 (51.4%) being male and 225 (48.6%) being female. It is worth noting that the Egyptian nationality was the most common nationality among the participants, a finding consistent with previous studies [15]. Furthermore, in our current study, there were more foreign pilgrims than domestic ones, which aligns with recent research [15].

A significant majority of pilgrims experienced MSK injuries, with 372 (80.3%) reporting such discomfort, particularly in the form of muscular pain (99, 58.6%), during the current Hajj season. This high percentage aligns with the results of prior similar studies, which also reported a prevalence exceeding 1,190 (80%) [12]. This study has roughly similar results to a previous study [12] in the site of injury, where the most common sites were in the legs and calves (117, 25.3%), foot and ankle (102, 22%), knee (65, 14%), arm (33, 7.1%), and lower back (28, 6%). Furthermore, in line with previous research, common comorbidities among pilgrims included diabetes and hypertension [15].

In this study, the most prevalent cause of injury was attributed to fatigue (206, 55.4%), followed by falls (76, 20.4%), crowding, road traffic accidents (RTA), and wheelchair-related accidents. This contrasts with a prior study, which suggested that foot twisting and falling were the most common causes of injuries [15]. This disparity may be attributed to the age group of the participants, as older individuals tend to experience fatigue more readily than their younger counterparts, exacerbated by the progressively rising temperatures each year [15]. Despite the passage of four years since the onset of COVID-19 and despite the relatively modest sample size for this year, overcrowding continues to be a contributing factor to injuries during the Hajj. This underscores the inevitability of overcrowding in large gatherings [15]. Unfortunately, the fracture rate in this study (97, 21%) has increased in comparison to the similar study mentioned previously, where it was only 13 (5%). This difference could also be explained by the age group of the participants.

To the best of our knowledge, this study stands as the first comprehensive examination of all MSK injuries that could potentially impact pilgrims during the Hajj season, encompassing a wider spectrum beyond mere pain or ligament tears. An inherent strength of our study lies in our approach to sourcing participants, as we collected data from a multitude of accessible gathering spots for pilgrims, not limited solely to hospitals.

This study is not devoid of limitations. Notably, our study did not include measurements of the participants' body mass index (BMI), despite previous research suggesting that BMI is a factor affecting the prevalence of MSK injuries [12]. Additionally, the study's reliance on a cross-sectional design restricts our capacity to delve deeper into exploring associations between variables. Furthermore, the relatively small number of participants in our study may hinder the generalizability of the results to a broader population with similar characteristics.

Similar to the recommendations made in prior studies [12,15], we suggest that future research endeavors consider conducting qualitative studies to delve into the associations between the type of injury and variables such as age and the site of injury. The use of translators in the data collection process could enhance accuracy and inclusivity. Furthermore, it is advisable to prioritize the education of pilgrims regarding the factors and risks associated with acquiring MSK injuries, with a particular emphasis on heightened awareness in crowded settings. This could be achieved through extensive awareness campaigns. Moreover, larger sample sizes in future studies would enhance the ability to generalize the findings to a broader population.

## Conclusions

The findings of this study highlight the common occurrence of MSK injuries among pilgrims during the Hajj season. Muscular injuries were the most frequently reported, particularly among young pilgrims, whereas joint injuries were more prevalent among older individuals. In terms of suggested methods to prevent MSK injuries among pilgrims participating in the Hajj season, the most commonly reported strategies included prioritizing rest and minimizing physical exertion.
